# Case Report: Visceral leishmaniasis misdiagnosed as systemic lupus erythematosus in a 36-year-old migrant worker

**DOI:** 10.3389/fmed.2025.1614790

**Published:** 2025-08-06

**Authors:** Li-Ping Sheng, Bo-Zhi Lin, Li-Na Han, Gui-Qiang Wang, Feng-Qin Hou

**Affiliations:** ^1^Department of Infectious Diseases and Center for Liver Diseases, Peking University International Hospital, Beijing, China; ^2^Department of Infectious Diseases, Peking University First Hospital, Beijing, China

**Keywords:** visceral leishmaniasis, systemic lupus erythematosus, liposomal amphotericin B, bone marrow, diagnosis

## Abstract

Visceral leishmaniasis (VL), also known as kala-azar, is an often-neglected tropical disease caused by *Leishmania donovani*. It is endemic in certain regions of China, including Shanxi Province. This case report describes a 36-year-old female migrant worker who regularly travels between Shanxi Province, her hometown and Zhongshan, Guangdong Province. She presented with prolonged fever, splenomegaly, pancytopenia and high title anti-nuclear antibodies. She was initially diagnosed with systemic lupus erythematosus (SLE) and associated polyclonal gammopathy. Her condition failed to improve with corticosteroids and immunosuppressants. Further evaluation revealed VL, confirmed by bone marrow smear and molecular testing. Treatment with liposomal amphotericin B led to prompt clinical resolution. This case highlights the importance of obtaining travel history and considering parasitic infections in patients with refractory fever and splenomegaly.

## 1 Introduction

Visceral leishmaniasis (VL), a protozoan infection caused by *Leishmania donovani complex*, represents a life-threatening tropical disease with over 95% mortality rate in untreated cases and an estimated 50,000 to 90,000 new cases occur annually worldwide (1, 2). While the classic triad of chronic fever, pancytopenia, and hepatosplenomegaly can be revealing in endemic regions, these findings can also occur in systemic lupus erythematosus (SLE) (3–10). Moreover, active VL’s ability to mimic SLE by producing antinuclear antibodies (up to 82% in recent series) and other autoantibodies in active VL (11) contributes to the overlap between these two disorders and diagnostic pitfalls. We present an illustrative case representative of a group of population, namely migrant worker, who regularly travels between Shanxi Province, China, a well-documented endemic region for VL (12) and her workplaces, Zhongshan, Guangdong Province, where VL is rare. Substantial descrepancies in febrile disease spectrum between endemic and non-endemic areas, poor coverage of routine laboratory studies for leishmaniasis, failure to ellucidate full travel history, as well as over-reliance of auto-antibody positivity might contribute to the diagnostic delay. The patient’s 9-month diagnostic odyssey, involving multiple, escalating immunosuppressive regimens with subsequent clinical deterioration, underscores the vital importance of detailed travel history, knowledge of VL as a “great masquerader” in autoimmune-like presentations and targeted parasitological evaluation in chronic febrile pancytopenia patients unresponsive to corticosteroids.

## 2 Case presentation

**Figure 1:**
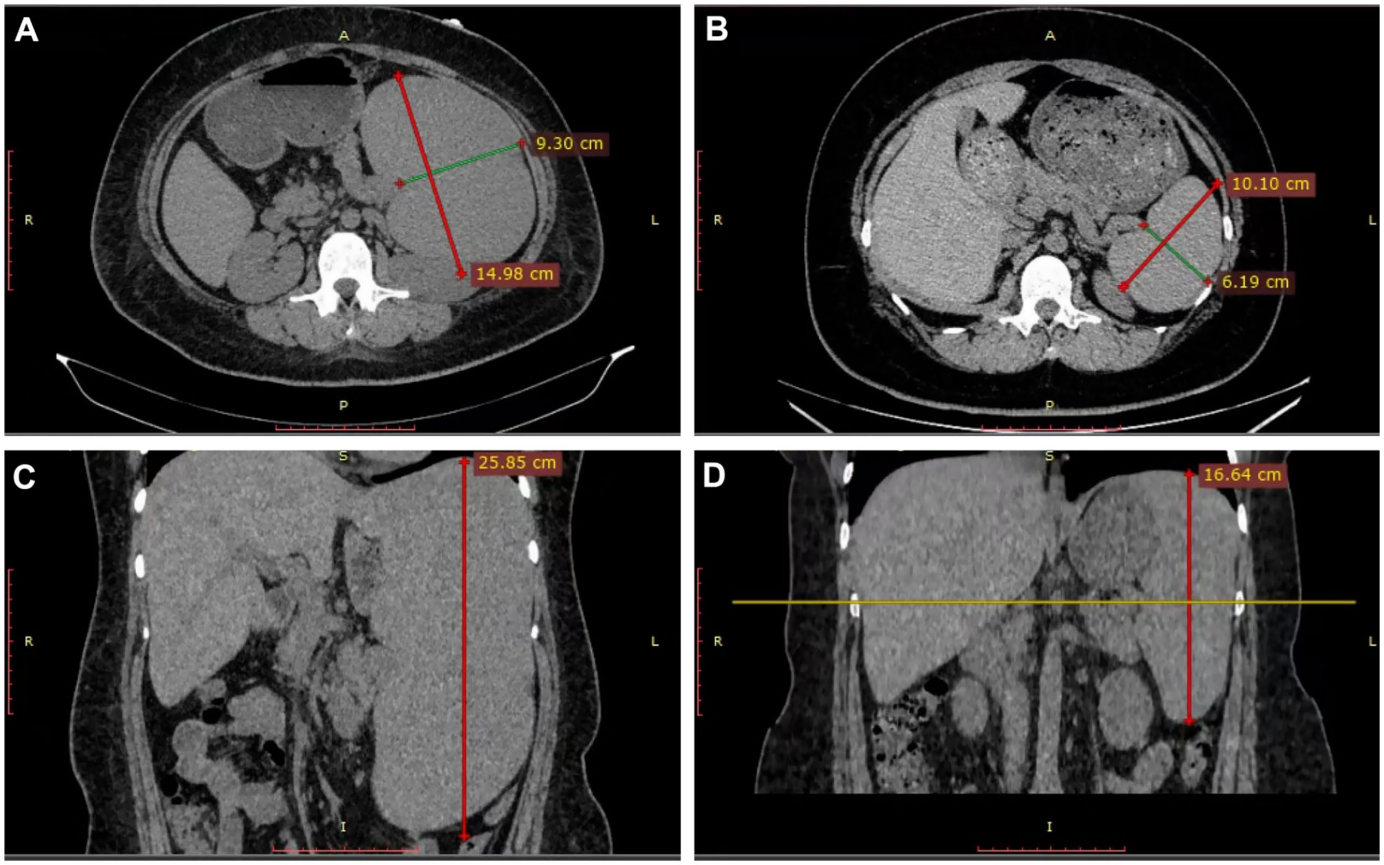
Non-contrast CT before **(A,C)** and 4 months **(B,D)** after liposomal amphotericin B therapy. Measurements were made at the level of splenic hilum.

**Figure 2:**
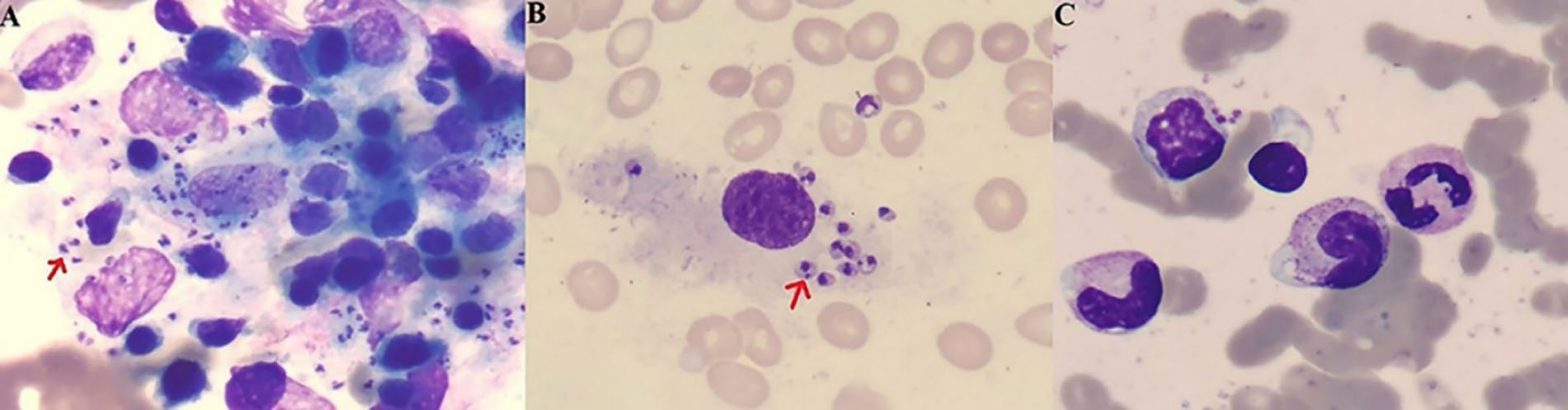
Bone marrow smears (Giemsa stain) showing intracellular Leishmania parasites at 400 × **(A)** and 1000× **(B)** magnification. No parasites were detected in post-therapy bone marrow cytology at 1000× magnification **(C)**. Methods of specimen preparation, staining followed Chinese Center for Disease control and prevention (CCDC) recommended standards for detection of Leishmania - Smear microscopic exanimation (WS/T 10018-2024) (https://cspd.ipd.org.cn/standardv138.html).

**Figure 3:**
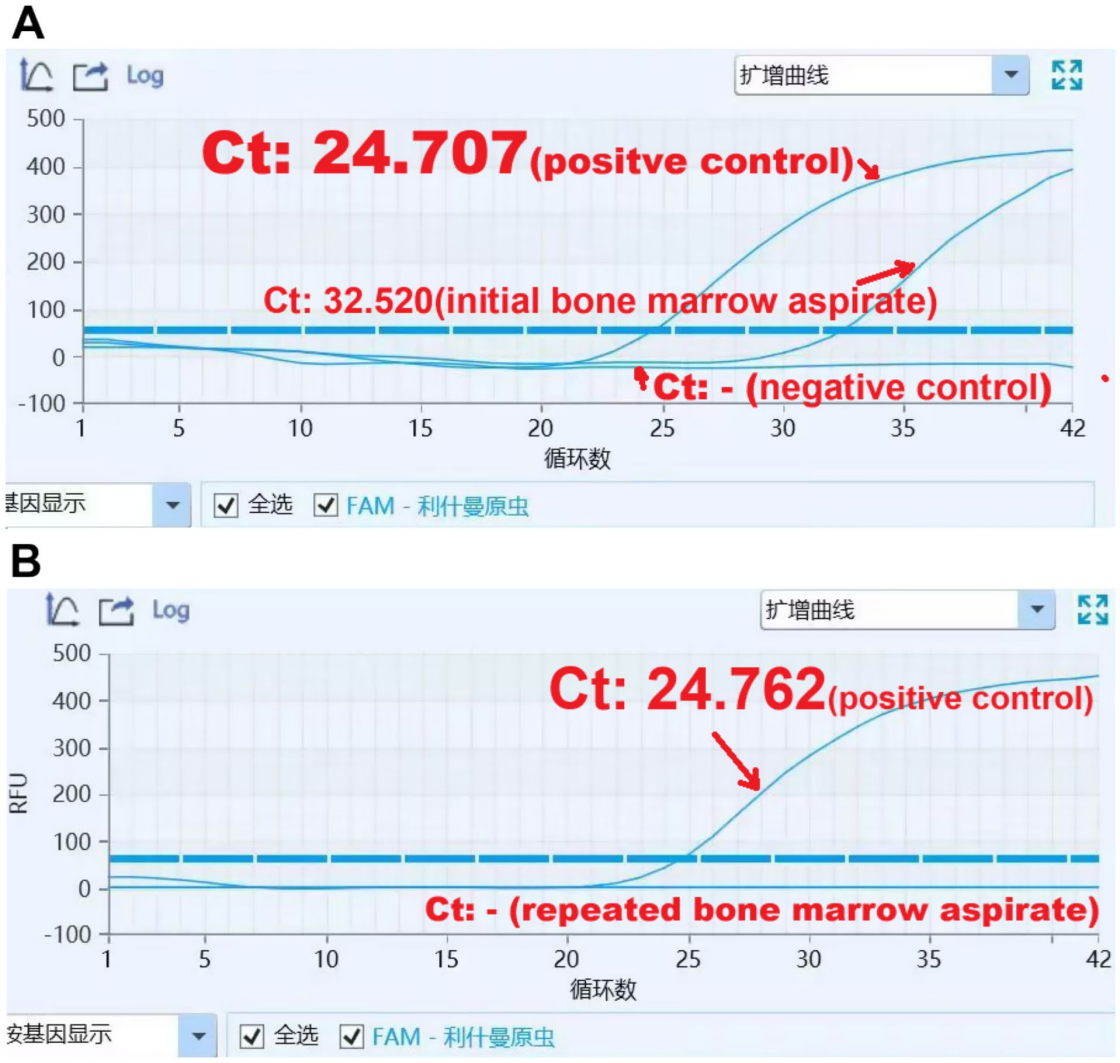
Real-time PCR amplification plots for Leishmania spp. DNA detection, both in pre- 203 treatment bone marrow aspirate sample **(A)** and post-treatment sample **(B)**. Also shown are 204 positive and negative control and corresponding Ct (Threshold Cycle) value.

A 36-year-old woman migrant worker from Shanxi Province, working and residing in Guangdong Province with her husband, presented with a 9-month history of fever, fatigue, joint swelling and weight loss. Other medical history is unremarkable except a 4-year history of untreated asthma. She denied contact with animals or febrile patients. Her husband have been otherwise healthy. No family history of susceptibility to recurrent infection were documented. She was initially evaluated at a local hospital in Zhongshan City. Laboratory tests revealed hemoglobin of 91 g/L, white-cell count of 2 × 10^9/L, platelet count of 73 × 10^9/L, elevated inflammatory markers [C-reactive protein (CRP), 35 mg/L; erythrocyte sedimentation rate (ESR), 59 mm/h; ferritin, 256 ng/mL], and positive antinuclear antibodies (ANA, titer 1/320) and anti-mitochondrial antibodies (AMA-M2). Serum IgG was markedly elevated at 53 g/L. Liver, renal functions and Immunofixation Electrophoresis (IFE) of Blood and Urine were normal. Bone marrow examination reportedly revealed hypercellular marrow with erythroid hyperplasia, with no evidence of malignancy or parasites. Abdominal computed tomography revealed hepatosplenomegaly. Her symptoms worsened, and she subsequently experienced high spike fever up to 40°C, profound fatigue, and significant weight loss. She was admitted to a rheumatology department in a tertiary hospital. Laboratory results confirmed pancytopenia, polyclonal gammopathy, and more importantly, revealed multiple auto-antibodies (ANA, titer 1:320, anti-dsDNA and anti-PM-Scl antibodies), positive direct Coombs test (DCT) and hypocomlementemia (low serum C3 and C4 level) (Table 1). Abdominal ultrasound reported hepatosplenomegaly, with the spleen measured 25 cm at long axis. Repeat bone marrow smears and biopsy showed no further diagnostic clue, reportedly. A presumable diagnosis of SLE was made. She then received high-dose glucocorticoids, cyclosporine, hydroxychloroquine, tofacitinib, and telitacicept (a BLyS/APRIL inhibitor). But her fever persisted with low level grade and pancytopenia showed minimal improvement. Ten days after self-discontinuation of prednisone (previously tapered to 20 mg daily) and cyclosporine (50 mg twice daily), the patient developed recurrent high spike fever, prompting her to our department. After reviewing her disease course, especially poor responsiveness to intenisve immunosuppressive therapy, a more targeted history taking and diagnostic work-up have been made, with a high prior suspicion of visceral leishmaniasis. Laboratory investigation confirmed anemia, leucopenia, and thrombocytopenia, increased ESR, CRP, ferritin and IgG (Table 1). Non-contrast computed tomography showed marked hepatosplenomegaly (Figures 1A,C). Definitive diagnosis was made through direct visualization of intracellular *Leishmania donovani* parasites in smear of bone marrow aspirate (Figures 2A,B), which was further confirmed by the Chinese Center for Disease Control and Prevention (CCDC). Molecular confirmation was also achieved by positive Leishmania-specific PCR (Figure 3A). Treatment with liposomal amphotericin B (L-AMB, 1.3 mg/kg daily) (13) resulted in rapid defervescence within 48 h. gradual improvement in hematologic parameters (Table 1) and regression of hepatosplenomegaly. Three-week post-treatment bone marrow detected no parasites (Figure 2C) and negative Leishmania PCR (Figure 3B). Pharmacological therapy was prolonged to 30 days given relapse concerns. Discharged on March 2025 in asymptomatic condition, the patient underwent scheduled outpatient follow-up with serial monitoring. At the 3-week post-treatment evaluation, laboratory investigations revealed complete normalization of peripheral blood cell counts (Table 1). The most recent abdominal computed tomography, 4 months after L-AMB infusion demonstrated significant regression of splenomegaly (Figures 1B,D). These objective findings confirm a favorable hematologic and clinical response to the therapeutic regimen. Continued surveillance is recommended to assess long-term disease resolution and monitor potential late complications.

**Table 1:** Laboratory data.

Variable	Reference range	Rheumatology department, other hospital Day 1	Rheumatology department, other hospital Day 45	This hospital Day 1	This hospital Day 30	21 Days after discharge
Hemoglobin (g/dl)	115–150	63	74	59	99	117
Hematocrit (%)	35–45	22	23	18.8	30.6	40
Leukocytes(×10^9^/L)	3.5–9.5	0.77	0.9	0.87	2	4.4
Differential (%)						
Neutrophils	1.8–6.3	0.34	0.4	0.47	0.9	2.3
Lymphocytes	1.1–3.2	0.27	0.4	0.32	0.86	1.6
Eosinophils	0.02–0.52	0	0.01	0	0.01	0.03
Platelets (×10^9^/L)	125–350	63	71	29	143	170
CRP (mg/L)	≤10	28	66	44.5	2.6	
ESR (mm/1st h)	0–20	119	80	84	87	87
Ferritin (ng/ml)	4.63–204	354		1,007	100	
LDH (U/liter)	120–250	Negative		284		
AST (U/liter)	13–35	12	8	23	19	
ALT (U/liter)	7–40	5	6	7	11	
Albumin (g/l)	40–55	25.2	30	20	31.8	
Scr (umol/L)	45–84	78		80	74	
D-Dimer (ng/ml)	≤250	Negative		3,936	154	
ANA (titer)	<1:40	Positive		1:160	1:160	
RF(IU/ml)	0–20	12		Not done		
Anti-Sm antibodies		Negative		Negative		
Anti-dsDNA antibodies		Positive		Negative		
Lupus anticoagulant		Negative		Not done		
Direct coombs test		Positive		Positive		
AMA-M2		Positive		Not done		
Complements						
C3 (mg/dl)	0.9–1.8	0.6		0.56	0.81	
C4 (mg/dl)	0.1–0.4	Negative		0.18	0.15	
IgG, mg/dl	7–16	39	32	41.7	42.7	45
Viral hepatitis B and C		Negative		Negative		
Cytomegalovirus		Negative		Negative		
Epstein–Barr virus		Negative		Positive		
HIV		Negative		Negative		

CRP, C reactive protein; LDH, lactate dehydrogenase; AST, aspartate aminotransferase; ALT, alanine aminotransferase; Scr, serum creatinine; ANA, antinuclear antibodies; RF, rheumatoid factor; Sm, Smith; AMA-M2, mitochondrial M2; IgG, immunoglobulin G; HIV, human immunodeficiency virus.

## 3 Discussion

This paradigmatic case highlights the critical diagnostic challenges posed by visceral leishmaniasis (VL) in patients presenting with autoimmune-like features. Well-documented cases (3–10, 14–16) where VL was initially misdiagnosed as systemic lupus erythematosus (SLE) have been previously reported, mainly in endemic regions. Despite diagnostic challenges due to the significant overlap between VL and SLE, lessons learned in the pesent case can be summarized as follows: First, the presence of massive splenomegaly (spleen length >20 cm in our case) should often raise suspicion of etiologies other than SLE, as this degree of organomegaly is exceptionally rare in SLE (5). Second, pancytopenia, hypocomplememia, direct Coombs’ test and high-titler anti-nuclear antibodies, as observed in our patient does not neccesarily gurrantee the diagnosis of SLE. Over-relience on these non-pathognomonic findings might be misleading. Alternative explainations, e.g., hypersplenism, should always be checked before these manifestations can be attributable to SLE. Inadequate response to multiple immunosuppressive regimens including high-dose corticosteroids and disease-modifying agents strongly argues against primary autoimmune pathogenesis. Third, careful history-taking, especially thorough evaluation of residence, travel and work locations provides crucial clue to often overlooked infectious etiologies. At first look, the patient’s disease presentation at non-endemic areas, i.e., Guangdong Province, might be an obstacle for considering endemic diseases like VL. A full travel history revealed her hometown, Shanxi Province, which accounts for nearly 38% of China’s reported cases (12). Patients presenting with “SLE features” plus splenomegaly or refractory cytopenia should undergo parasitological evaluation.

Current Infectious Diseases Society of America (IDSA) and WHO treatment guidelines (2, 17) uniformly recommend liposomal amphotericin B as first-line therapy, reflecting its superior efficacy and safety profile. Our patient’s excellent response to this regimen, despite the 9-month diagnostic delay, reinforces these evidence-based recommendations while underscoring the remarkable reversibility of even advanced VL with appropriate antimicrobial therapy. Given the prolonged disease duration and pre-existing immune dysfunction secondary to corticosteroid and immunosuppressant therapy, the treatment course was extended to 30 days to mitigate the elevated risk of disease recurrence (13).

This case serves as a poignant reminder that in tropical medicine, the presence of autoantibodies never trumps fundamental clinicopathological correlation. When epidemiological risk factors, characteristic physical findings, and atypical disease course collectively point toward infectious etiology, physicians must have the courage to question autoimmune diagnoses and pursue definitive microbiological confirmation - approach that ultimately saved this patient’s life. A major limitation of this case was our inability to investigating and reconstructing a complete epidemiological time-line, given the sporadic nature of the present case. High prevalence of asymptomatic leishmaniasis carriers (18) further compromise our ability to delineate exact source of transmission in our particular case. Based on the regional distribution of leishmaniasis in China (19), we speculated that the most probable source were from contact with vector insects or asymptomatic carriers in endemic regions, i.e., Shanxi Province in our case.

In conclusion, VL should be considered when patients present with “autoimmune” features refractory to therapy. Full travel history for migrant workers is essential in elucidating key epidemiological clues. This case highlights the importance of considering VL in the differential diagnosis of patients with prolonged fever, hepatosplenomegaly, and pancytopenia, especially in endemic areas. Early diagnosis and treatment are crucial for improving patient outcomes. Further studies are needed to improve the awareness and diagnostic capabilities for VL in non-endemic regions.

## Data Availability

The raw data supporting the conclusions of this article will be made available by the authors, without undue reservation.
